# Cancer education in Ukraine

**DOI:** 10.3332/ecancer.2013.369

**Published:** 2013-10-31

**Authors:** Vasyl F Chekhun, Iryna V Shepelenko

**Affiliations:** RE Kavetsky Institute of Experimental Pathology, Oncology and Radiobiology of National Academy of Sciences, Kyiv, 03022, Ukraine

**Keywords:** oncology, high medical education, teaching, training

## Abstract

The main features of the system of ‘higher medical education’ in Ukraine are presented. The principles of undergraduate, specialist training, and postgraduate education on oncology are described in detail and discussed in terms of European standards of cancer education. It is underlined that the cancer education in the system of higher and postgraduate education should be continuous, multidisciplinary, and of high quality.

During the last two decades in Ukraine, there have been significant changes in the field of higher education as a whole as well as in the medical field. They have affected the philosophy of teaching and training, its global goals and objectives, organisational structure, the content of education, approaches to the development of educational standards and curricula, forms and methods of quality assurance, and many other components of the educational process. The national public health strategy, a multi-sectoral comprehensive programme ‘Health of the Nation’, is aimed at a wide spectrum of critical challenges to public health and health care—improving the teaching and training of health professionals is one of its goals.

The development and reform of the Ukrainian national health system needs to prepare a new generation of highly skilled health-care professionals. The new concept of higher medical education (HME) was adopted in Ukraine some years ago. The term ‘high education’ means the level of knowledge acquired in higher education institutions (university) on the basis of general secondary education; it is a result of a consistent, systematic, and deliberate process of mastering educational content and is completed via obtaining a specific qualification on the basis of state certification, and an individual who successfully passed the state certification receives a document on higher education. This new concept of HME is aimed at improving the quality of medical specialists, integrating of medical education and science, solving staff problems, improving health care, and ensuring the competitiveness of university graduates in the domestic and international labour markets [1]. This concept is based on modern requirements for the level of quality in HME, dynamic changes in the national health as well as social, economic, ethical, and legal characteristics of the activities of the health system in Ukraine, development of medical science and evidence-based medicine, and intensification of international cooperation in the field of medical education. The concept’s implementation is expected to create legal, economic, and organisational conditions for improving the quality of HME by aligning its standards to international standards and make efficient use of materials and technical and scientific potential.

We need to create an efficient system of continuing medical education, which is logically replaced with the term ‘continuous professional development’, meaning a period of education and training of physicians, which begins after the completion of basic medical education and postgraduate training, and continues thereafter throughout the whole professional life of every physician. Continuing professional development is regarded as a professional obligation of every physician and as education that is on-going in the form of self-study but not under anyone’s leadership. If basic medical education and postgraduate training are appropriately regulated and formalised, for continuing professional development every physician is personally responsible.

The reformation of HME was initiated soon after Ukraine gained independence, and work is now under way to assess training programmes and to bring them in line with European standards [2]. The system of medical education in Ukraine as a whole is as follows: state policies stipulate that HME shall remain in the state health system. HME is provided, mainly in state-owned university-level medical schools and faculties as well as postgraduate training institutions. Standards for HME as well as curricula and qualification requirements for specialist training are set by the state. HME is organised into several stages, comprising generalist medical education, specialist training, and postgraduate training at the master of science level.

At present, education is provided by 19 state medical universities and three postgraduate medical academies. The universities are funded by the Ministry of Health and are supervised by both the Ministry of Health and the Ministry of Education & Science. In addition, there are three medical institutes within multi-speciality universities supervised and funded by the Ministry of Education & Science. Figure 1 presents the general principles of medical education as a whole and education on speciality oncology as well. All of them are financed from the state budget, but they also have their own finances due to the education of 40%–50% students being on a so-called contract basis (they have to pay for their education themselves). These students are citizens of Ukraine as well as other countries. Each of these 22 medical universities or institutes has a Department of Oncology.

Graduate medical education aims to train general practitioners, with training usually lasting for six years. Medical students have to complete two state licensing examinations during their undergraduate training, after studying basic disciplines and after completion of the full training course. Postgraduate medical training is based on the principle of continuous education and involves a main specialisation, further specialisation, and the advanced professional training of physicians. The main specialisation is achieved through an internship that combines full-time and extracurricular forms of training.

Why is it such an important and urgent problem for Ukraine to improve and/or modify the system of cancer education?

There are many reasons for this, and the main one is the fact that the levels of cancer caused morbidity and mortality in Ukraine are still growing. This tendency coincides with the state of art in most European countries or in the United States. During the last decade, the cancer morbidity in Ukraine has increased, and according to the data of the State Statistic Service of Ukraine, the number of newly registered cases increased from 382,000 in 2000 to 433,000 in 2012 [3]. According to the National Cancer Register, in 2011, the cancer incidence and mortality crude rates in Ukraine were correspondingly 370.7 and 186.3 per 100,000 of population [4]. Figure 2 represents the relative frequency of ten leading cancer site incidences and mortality in Ukraine in 2011 [4]. We must take into account that our population is nearly 47 million, and the number of practising medical oncologists is 4.3 per 100,000 of population [3].

Given the social importance of the problem of malignant tumours, we are convinced that the teaching of oncology should be a priority in the system of HME and in postgraduate training, so we must have highly skilled specialists in oncology. The teaching of oncology in the system of higher and postgraduate education should be continuous, multidisciplinary, high quality and effective.

The vertical system of cancer education in high medical schools (universities and institutes) has some stages (Figure 3).

The first stage lasts six years. The first two years are devoted to fundamental subjects that are common to all future physicians. They are anatomy, cytology, histology, biochemistry, normal and pathology physiology, and so on. During the next four years, our medical students receive education in clinical disciplines—oncology and medical radiology among them. In our high medical education system, these two specialities are separate, and education and training are performed consequently. According to the programme, medical radiology is taught in the fourth year and oncology in the fifth year.

For the subject ‘Oncology’, 90 hours is given (Tables 1 and 2) [5].

The second stage lasts two or three years. This is the period when our medical students are trained in internships and/or masters. The duration depends on the type of specialisation that they chose. If it is therapeutic specialisation, it is two years; if it is surgery, then it takes three years.

A characteristic feature of this form of training is to enable young interns to have the maximum independence in their work. However, they are responsible for expanding their knowledge of diagnosis and relationships with patients in the treatment process. A young doctor working in hospital wards is responsible for the patients, for the design of the hospital’s patient’s case records and other medical records, he is directly involved in all aspects of patient examination and, if it is an internship in surgery, in operational activities. It is important that young doctors do not have the responsibility of personal decision making as they will always be working with supervisors who are responsible for the diagnostic and treatment process.

After this training, the students receive their speciality. In our case, this is physician oncologist. Along with this, they also receive the right for their own practical clinical activity as a doctor of the third category. Some years ago in Ukraine, there were only three educational qualifications of master’s and specialists in oncology—namely, surgeon oncologist, child oncologist, gynaecologist oncologist. There are now three new qualifications: physician cytologist, chemotherapeutical physician, and a very wide speciality—clinical oncologist.

The next step is clinical residency training or graduate study. It is not obligatory, but our young specialists are interested in such additional training. This is due to the fact that they can obtain more practical skills (the first case) or obtain a PhD—this is the second variant.

It must be emphasised that all the years during which our students are studying and training in clinical subjects are spent in a hospital setting. Applying to the oncology speciality, they study at regional or inter-regional cancer centres. There are 27 such centres in Ukraine. They have all or nearly all the facilities for diagnosis and treatment of cancer patients. In recent years, these centres have received modern equipment for radiotherapy, molecular diagnosis, and so on.

The number of students who receive the speciality in oncology is not more than 5% of all those who graduate from all medical universities. Of course, we do not need many oncologists in our country, but not all of these young specialists will actually practice in oncology. While the number of cancer patients does not decline, we must have more physicians. First of all, it concerns family physicians who are at the forefront in newly diagnosed cases of cancer patients. In countries with large economies and where medicine is well financed, it is not a problem, but for our country where the financing of medicine still remains low, we are facing the problem of under-diagnosis of cancer cases at the early stages.

The next stage of medical education and training is postgraduate education. Postgraduate education in Ukrainian law is defined as the improvement of education and specialised training of a person through the deepening, expansion, and renovation of their professional knowledge, skills, or gaining another qualification on the basis of prior education, skill level, and experience. According to this definition, postgraduate education creates conditions for the continuity of education and includes *training *(gaining another speciality on the basis of the above-obtained education and skill level and experience); *specialisation *(acquisition by a person of abilities to perform certain tasks and responsibilities, which have specific features within the profession); *extension profile (skills development) *(acquisition by a person of the ability to perform additional tasks and responsibilities in the speciality); and *traineeships *(acquisition by a person of experience for performing the tasks and responsibilities of a particular speciality). A person who has successfully completed training or specialisation or extended profile (increased skills) receives a document of postgraduate education.

In Ukraine, there are three postgraduate medical academies with five departments of oncology; among these, there are two faculties of oncology, one of paediatric oncology, one of oncological surgery, and one of oncological gynaecology (Figure 1). 

Physicians who have completed formal medical training are required to pursue continuing professional development to maintain knowledge and skills, with corresponding programmes being provided at postgraduate medical faculties. All practising physicians are subject to regular attestation at a maximum of five years. Eligible physicians are required to have completed a pre-attestation cycle within one year before the official attestation, which is performed by committees at the Ministry of Health or regional health bodies. Moreover, such training is mandatory to all practitioners notwithstanding their place of occupation: either they work in clinics or in research institutes or educational universities.

Concerning postgraduate education and training, we must not forget about such mechanisms as international mobility. We consider this also to be self-education. It is one of the most effective instruments to provide opportunities to physicians to improve their skills. However, it is not very typical for Ukrainian physicians. About 10% of our oncologists are able to make training visits to leading hospitals in Europe for one to three months. This can only be done through grants. The fact that Ukraine is not even an associate member of the European Union makes it extremely difficult to obtain such an opportunity.

One of the main tasks of the Organisation of European Cancer Institutes (OECI)’s Working Group Education and Training (E&TWG) is as follows: ‘Through the E&TWG’s activities, the OECI hopes to create a European Area for E&T in oncology to tackle fragmentation in training, share knowledge and facilities among Cancer Centres, facilitate the launch of new research initiatives, reinforce existing successful actions, improve knowledge on new emerging fields, and create possibilities for the exchange of personnel between Cancer Centres and industries’. Taking this into account, we can expect that our partnership with this WG will make it much easier for young Ukrainian oncologists to have the opportunity for international mobility.

The courses that the OECI E&TWG organises on a continual basis concerning different issues of cancer diagnosis and treatment are extremely important for our oncologists, both practitioners and researchers, and the possibility of participation in such events will undoubtedly be useful for obtaining new current knowledge on very specific areas of oncology. We hope that the activities involved in designing the road-map for the participation of our young specialists in these events are completed on time. As is evident, the main problem is, as always, funding, after this issue is solved then the attendance and active participation of our young oncologists will be high, and the knowledge and skills that will be obtained during these courses will be implemented in the Cancer Centres of Ukraine.

In this context, it is necessary to emphasise a very exciting and extremely important moment, namely the decision to hold the 2014 OECIESO workshop in Ukraine (Kyiv) at the RE Kavetsky Institute of Experimental Pathology, Oncology, and Radiobiology, which is an OECI-associated member. This workshop will be devoted to the subject ‘Diagnostic Molecular Markers and Personalised Therapy in Breast and Prostate Cancer’. We are absolutely aware that it will open up new perspectives in sharing and improving knowledge on new trends in the field of oncology for our young specialists. We hope that it will be an example of close partnership between our Institute and the OECI E&TWG, which will facilitate the integration of cancer education/training system into Europe.

All our higher medical schools have now transferred to the so-called Bologna model of education. Nowadays, we are trying to assess the preliminary results of the implementation of this model. One of the very important disadvantages of the Bologna model is the absence of objective evaluation of the students’ level of knowledge, for example, the possibilities for simply ‘guessing’ while testing and the difficulties in identifying those students who have systemic knowledge. The teaching process is transformed into an incredible amount of papers, with calculation of a lot of digital indicators that are included in these papers, the transfer of assessment of learning from teachers to computers and partition of the object into fragments without further systematisation. But where is the attachment of each student to the tutor/consultant who will help him to compose and sustain an individual training plan? Where is the student’s independent choice of terms and sections of the studying program according to the system of: a) mandatory specialized subjects, that are adjustable in terms of studying by the administration of the university, b) mandatory subjects, that are studied in terms which are selected and agreed with the consultant, and c) elective subjects, that are of interest to the student but are chosen with the tutor’s help?

Ensuring continuity of higher education and training throughout life is an urgent problem that requires, above all, the improvement of training and the methodological support of cancer education in accordance with international requirements.

The key points for solving this problem can be broken down into a number of organisational, tactical, and strategic issues.

The first group, the organisational issues, is composed of those that could be solved during one to two years, for example: (a) creation of an electronic database ‘Oncology Departments of High Medical Schools’, which will include all the issues concerning the teaching process (from the management of the department to the material status of training facilities and provision with learning materials and so on); (b) evaluation of the needs of the Departments of Oncology for training materials, visual aids, and the planning of centralised production of educational materials; and (c) development of the standard for educational-methodical complex in oncology.

The second group consists of tactical perspective issues, the resolution of which might take two to three years. Among them are (a) all Departments of Oncology should use the same clinical classification of diseases, stages of morphological data, and so on, which will unify the teaching and reach the final standard of training oncologist; (b) development of so-called ‘schemes and standards of diagnosis and treatment in oncology’ aimed at the level of university students who will be characterised by some generalisation and less detail, so that after graduation, the student will be prepared to accept any kinds of standards—both local and state or international; (c) to form a team of authors for preparing a new basic textbook of oncology and publish this agreed textbook for high medical schools; and (d) to issue multimedia lectures on the basis of a new textbook, and professors should give lectures on the same methodology and content.

The third group comprises strategic, long-term issues, the resolution of which will take up to five years; they are (a) creation of an enterprise centralised production of visual aids in oncology for Ukrainian universities (slides, tests, lectures, glossaries and atlases, educational films, and so on); (b) changing the curriculum in the speciality ‘oncology’ (up to 120 hours instead of 90 at the medical faculty); (c) justification of the necessity to organise internship training in oncology and preparation of a methodological framework for its implementation in the Ministry of Health; (d) three years training in clinical ordinature on oncology based on cancer centres within the Departments of Oncology.

This is not a definitive list of issues and questions to be addressed, but these are the ones that are urgent and could be solved without significant financial support.

## Conclusion

The Ukrainian system of cancer education has finished its period of transition, but many specific urgent problems are waiting for solutions. Nevertheless, it has positive elements that are the basis for teaching and training our specialists in oncology to be highly skilled. We are absolutely sure that if policy makers and top managers in the health-care system would persist on one strategic line—implementation of the best world experience and accommodation of it to Ukrainian traditions—the results will be successful. Working together in close cooperation with medical education systems and models from many different countries, we can formulate the most optimal and effective system aimed at preparing the new generation of specialists in oncology that will meet the challenges of the 21st century.

## Figures and Tables

**Figure 1.**
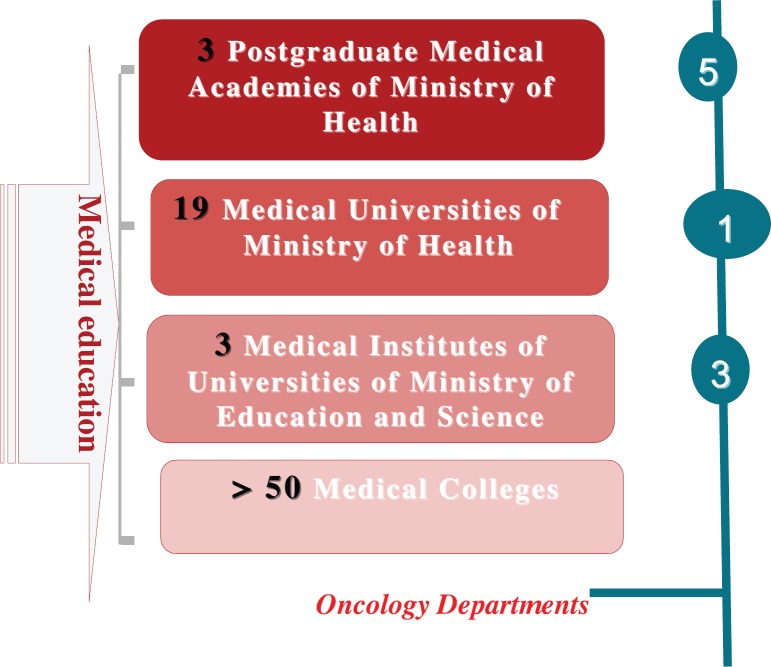
The system of medical education in Ukraine.

**Figure 2.**
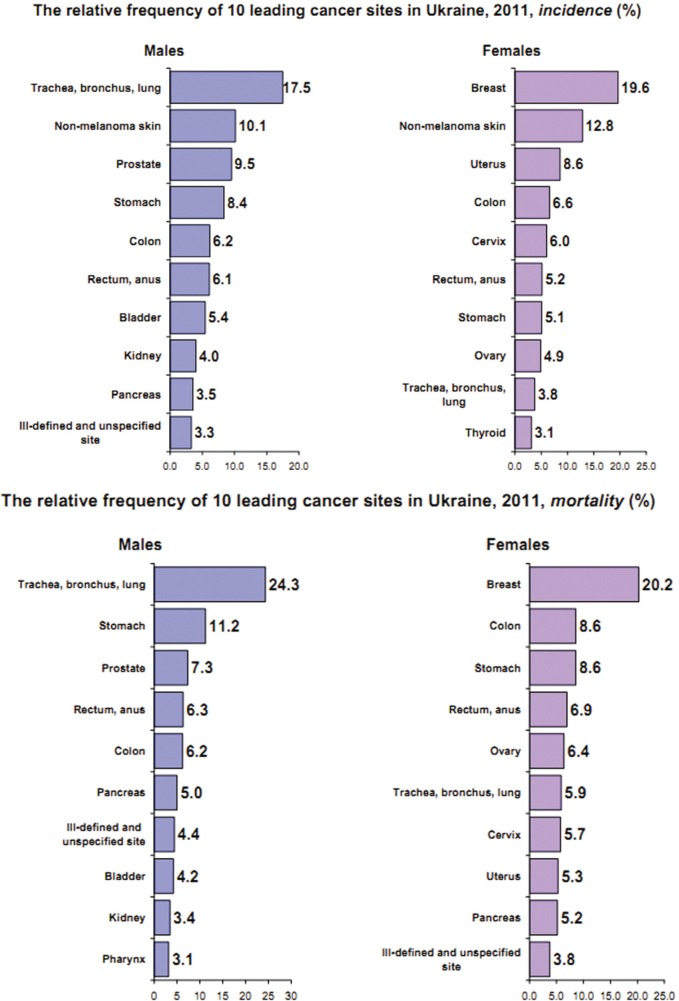
The relative frequency of ten leading cancer site incidences and mortality in Ukraine in 2011 (data from the National Cancer Register).

**Figure 3.**
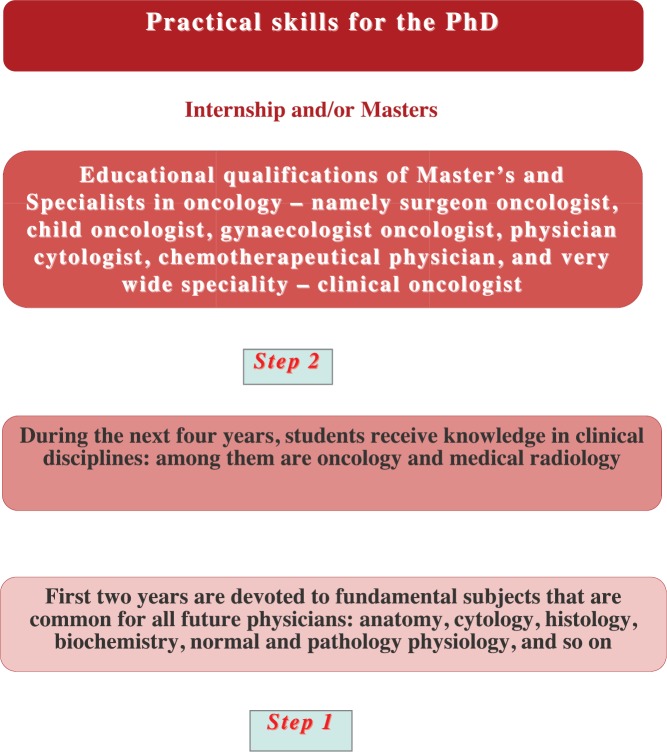
The vertical system of cancer education in medical universities in Ukraine.

**Table 1.** Distribution of 90 hours given to the subject ‘oncology’.

Oncology	Hours			
	Hours whole/credits	Auditor		Students self-study
		Lectures	Practice	
Total	90/3	20	50	20

**Table 2.** Programme on ‘oncology’ approved for medical universities.

Topic	Lectures	Practice	Students self-study
Oral cavity, oesophageal, stomach, hepatic, pancreatic, and colorectal cancers	7	19	5
Lung, breast, thyroid, skin cancers, melanoma, lymphomas, lymphogranulomatosis	6	13	5
Kidney, bladder, prostate cancers, and gynaecological cancers	4	9	3
Early diagnosis, cancer primary and secondary prevention; palliative and symptomatic therapy	3	14	7
